# Meta-Analysis of Tourette Syndrome and Attention Deficit Hyperactivity Disorder Provides Support for a Shared Genetic Basis

**DOI:** 10.3389/fnins.2016.00340

**Published:** 2016-07-22

**Authors:** Fotis Tsetsos, Shanmukha S. Padmanabhuni, John Alexander, Iordanis Karagiannidis, Margaritis Tsifintaris, Apostolia Topaloudi, Dimitrios Mantzaris, Marianthi Georgitsi, Petros Drineas, Peristera Paschou

**Affiliations:** ^1^Department of Molecular Biology and Genetics, Democritus University of ThraceAlexandroupolis, Greece; ^2^Laboratory of General Biology, Department of Medicine, Aristotle University of ThessalonikiThessaloniki, Greece; ^3^Computer Science Department, Rensselaer Polytechnic InstituteTroy, NY, USA

**Keywords:** Tourette Syndrome, ADHD, meta-analysis, cross-disorder, TBC1D7, GUCY1A3, RAP1GDS1, CHST11

## Abstract

Gilles de la Tourette Sydrome (TS) is a childhood onset neurodevelopmental disorder, characterized phenotypically by the presence of multiple motor and vocal tics. It is often accompanied by multiple psychiatric comorbidities, with Attention Deficit/Hyperactivity Disorder (ADHD) among the most common. The extensive co-occurrence of the two disorders suggests a shared genetic background. A major step toward the elucidation of the genetic architecture of TS was undertaken by the first TS Genome-wide Association Study (GWAS) reporting 552 SNPs that were moderately associated with TS (*p* < 1E-3). Similarly, initial ADHD GWAS attempts and meta-analysis were not able to produce genome-wide significant findings, but have provided insight to the genetic basis of the disorder. Here, we examine the common genetic background of the two neuropsychiatric phenotypes, by meta-analyzing the 552 top hits in the TS GWAS with the results of ADHD first GWASs. We identify 19 significant SNPs, with the top four implicated genes being TBC1D7, GUCY1A3, RAP1GDS1, and CHST11. TBCD17 harbors the top scoring SNP, rs1866863 (p:3.23E-07), located in a regulatory region downstream of the gene, and the third best-scoring SNP, rs2458304 (p:2.54E-06), located within an intron of the gene. Both variants were in linkage disequilibrium with eQTL rs499818, indicating a role in the expression levels of the gene. TBC1D7 is the third subunit of the TSC1/TSC2 complex, an inhibitor of the mTOR signaling pathway, with a central role in cell growth and autophagy. The top genes implicated by our study indicate a complex and intricate interplay between them, warranting further investigation into a possibly shared etiological mechanism for TS and ADHD.

## 1. Introduction

Gilles de la Tourette Syndrome (TS) is a childhood onset neuropsychiatric disorder characterized by a multitude of motor and vocal tics that last longer than a year. Its international prevalence has been estimated to be approximately 1% (Robertson et al., 2009). A recent systematic review and meta-analysis on the population prevalence of TS, refined its prevalence estimate in children to 0.3–1% (Scharf et al., 2015). It presents a significant gender bias, with 73% of its patients being male, and the male patients being more likely to develop comorbid disorders (Robertson et al., 2015). TS is often associated with other neuropsychiatric disorders, including Attention Deficit/Hyperactivity Disorder (ADHD), Obsessive Compulsive Disorder (OCD), depression and anxiety (Robertson, 2006).

The first genome-wide association study (GWAS) on TS was undertaken by the Tourette Syndrome Association International Consortium for Genetics (TSAICG) (Scharf et al., 2013). In their primary analysis, no SNPs achieved an association *p*-value of genome-wide significance, however this study provided the basis for subsequent studies, as the top signals that attained a *p* < 10^−3^ were found to be significantly enriched for functional variants. The Gilles de la Tourette Syndrome GWAS Replication Initiative (GGRI) undertook a replication (Paschou et al., 2014) of the first GWAS study, by selecting the top LD-independent SNPs and additional SNPs singificantly enriched in eQTL or mQTLs for genotyping in 609 European TS patients and 610 ancestry-matched controls, recruited from different European countries and Canada. This replication study enriched the significance of the selected SNPs and provided more evidence toward the genetic aetiology of TS.

On the other hand, initial GWAS attempts on ADHD also did not yield genome-wide significant results (Neale et al., 2008; Mick et al., 2010; Neale et al., 2010a; Lesch et al., 2008). To that end, a meta-analysis was conducted by Neale et al. (2010b), aggregating the results of the previous GWAS projects and meta-analyzing them. This meta-analysis could not produce any significant results either, but, similar to the TS GWAS, it set the groundwork for the elucidation of the genetic background of ADHD.

The relationship of TS with ADHD is well established (Karagiannidis et al., 2016). Individuals with ADHD commonly present tics, and in individuals with TS and tics, ADHD is a significant commorbidity. ADHD occurs in a significant proportion of TS patients, ranging from 21 to 90% in studied cohorts (Robertson, 2006). This phenotypic association is a major indication of a common genetic background between the two disorders. Furthermore, a recent study investigating the genetic correlation among neuropsychiatric and neurological disease based on GWAS results for each disorder, also recovered a genetic correlation between TS and ADHD (Anttila et al., 2016).

This is the first study to attempt to identify a shared genetic component between TS and ADHD. We used summary statistics from the latest large-scale genomic efforts to unravel the genetic background of TS and ADHD and derived the combined effects of shared polymorphisms between the two datasets, highlighting genes and pathways that may play a role in the shared etiology between the two disorders.

## 2. Materials and methods

### 2.1. Data sources

For our study we focused on the combination of the known effects of SNPs on the phenotypes of TS and ADHD.

Scharf et al in their study (Scharf et al., 2013) performed a GWAS and meta-analysis on a total of 1285 cases and 4964 ancestry-matched controls of European ancestry, genotyped on 484,295 SNPs. The dataset was analyzed in three split cohorts and was subsequently meta-analyzed. The study reported 552 SNPs associated with TS that acquired a *p* < 10^−3^.

We acquired the ADHD meta-GWAS whole-genome summary statistics from the study conducted by the ADHD subgroup of the Psychiatric GWAS consortium (Neale et al., 2010b; Cross-Disorder Group of the Psychiatric Genomics Consortium, 2013). The total sample size consisted of 896 cases, 2455 controls and 2064 trios genotyped and then imputed to 1,230,536 SNPs.

We used the publically available top SNPs with a *p* < 10^−3^ associated with TS (Scharf et al., 2013) and meta-analyzed them with the results of the ADHD meta-analysis (Neale et al., 2010b; Cross-Disorder Group of the Psychiatric Genomics Consortium, 2013). We identified 489 SNPs that were overlapping between the two sources to proceed with the meta-analysis.

### 2.2. Meta-analytical procedure

We combined the effects of the SNPs in each phenotype, following a meta-analytic approach, assuming a fixed-effects model, using the Z-Scores as the effect and the number of cases in each study as the weight. The heterogeneity of each analyzed SNP was assessed using Cochran's *Q*-test and *I*^2^ statistic. The analysis was performed using the METAL (Willer et al., 2010) software. The significance threshold was set using the Bonferroni correction for multiple testing.

### 2.3. Annotation and functional significance

We proceeded to analyze the significant SNPs using the ENSEMBL Variant Effect Predictor (McLaren et al., 2010) to annotate and explore the possible functional characteristics of the associated variants. The genomic positions of the variants were converted to the GRCh38 assembly coordinates. For the investigation of the allelic frequencies, and the linkage disequilibrium (LD) patterns we used data from the 1000Genomes project (1000 Genomes Project Consortium et al., 2015) and the LDlink software (Machiela and Chanock, 2014). To investigate the association of the variants and their respective genes with tissue expression levels we used the GTEx portal (The GTEx Consortium, 2013) and the Expression Atlas database (Petryszak et al., 2014). The annotation and exploration of the genomic structure of the identified loci was further assisted by the use of LdOOKUP, developed by Shaun Purcell (https://purces04.u.hpc.mssm.edu/ldookup/ldookup.cgi).

We have uploaded all codes necessary to confirm our conclusions and they can be found at https://github.com/ftsetsos/tsadhdmeta2016.

## 3. Results

The meta-analysis produced 19 significant SNPs, out of the total 489 tested. The significance threshold was set using the Bonferroni correction for multiple testing, setting the significance level at a *p*-value of 0.0001022.

Of these 19 SNPs, five attained the lowest *p*-values, coupled with no evidence of confounding heterogeneity (*I*^2^ = 0). The tested SNPs that present the most significant heterogeneity (Het *p* < 0.05) were the ones that had achieved a *p* < 0.05 in the original ADHD meta-analysis. The annotation showed that the majority of the significant variants are located in introns, two are in regulatory regions, while three are intergenic.

The first and third top hits (rs1866863, *p*:3.23E-07 and rs2458304, *p*:2.54E-06) reside on a LD-block of 54.19 kb on the 6p24.1 region. They show significant linkage disequilibrium between them (*D*′: 0.909, *R*^2^: 0.725). The former is a variant located in the regulatory region downstream of the TBC1D7 gene, and the latter is an intron variant in the same gene. Both are in LD with the rs499818 eQTL (*R*^2^: 0.59 and 0.48 respectively), suggesting an interplay with the expression levels of the gene.

Chromosome 4 hosts the second (rs2705462, *p*:1.44E-06), the fourth (rs17561798, *p*:9.89E-06), and two lower-ranked variants(rs477897, *p*:8.65E-05 and rs2285084, *p*:1.00E-04), each residing in four distinct, LD-independent loci. The most significant variant, rs2705462, is located in the intergenic region upstream of the GUCY1A3 gene in the 4q32.1 region on a LD-block of 46.63 kb. The next variant, rs17561798, resides in the 4q23 region and is an intron variant inside the RAP1GDS1 gene. The variant rs477897 is located within an intron of ADD1 in the region 4p16.3 captures an area of 125.59 kb, implicating the genes H3BP2, ADD1, MFSD10, NOP14. The intron variant rs2285084 is located in the gene ANXA10. The gene is included in the locus 4q32.3 in a high LD region of 330.00 kb that contains also the genes ANXA10, DDX60, DDX60L.

The fifth most significant SNP, rs1650137 (*p*:1.76E-05), is located on 12q23.3 in an intron of the gene CHST11. This region is inside a LD-block that extends for 39.68 kb. The variant rs2246417 came up as the sixth most significant (*p*:1.95E-05), residing in the locus 21q22.3 in a LD-block of 16.29 kb, within an intron of the LINC00316 gene.

The variant rs11716445 (*p*: 8.01E-05) resides in the 3p21.31 region. This region is characterized by a very large area with an extended high-LD block of 1941.64 kb and it contains 70 genes, with the first genes being PLXNB1, CCDC51, TMA7, ATRIP, TREX1, and ending with HYAL2, TUSC2, RASSF1, ZMYND10, NPRL2. The variant itself is located in the intron of the RHOA gene. It is one of the lower-ranked variants that achieved minimal confounding by heterogeneity.

The locus 7p21.3 hosts the intergenic variants rs13244651 (*p*:4.11E-05) and rs17531553 (*p*:7.08E-05) that are part of a LD-block sized at 103.93 kb, albeit with no known genes close to them, and no strong suggestive results for any direct functional implication.

Two genomic regions on chromosome 9 are implicated by our results. A region of 58.16 kb in the 9p24.2 locus contains the variants rs1007021 (*p*: 4.38E-05) and rs1007022 (*p*:7.68E-05) in the introns of KCNV2, showing strong LD between them (*D*′: 1.000 *R*^2^:0.803). The region 9q31.1 contains the intergenic variant rs7858600 (*p*:5.30E-05) inside a region of 27.35 kb.

In the locus 10q21.1, the intergenic variant rs1896373 (*p*:7.46E-05) captures a region of 47.58 kb, in strong LD with the rs1919459 eQTL (*R*^2^: 0.97) that is associated with the regulation of DKK1. The variant rs4789936 (*p*:8.92E-05) is located in the 17q25.3 locsus, in a LD-region 34.19 kb, and is an intron variant of the gene TIMP2 while on it is non-coding exon variant in the gene CEP295NL. In the locus 16q12.1, the variant rs7203818 (*p*:1.01E-04) resides in a LD-block of 21.12 kb within an intron of ZNF423.

On chromosome 13, the LD-associated variants rs7336083 and rs9319159 (*D*′: 0.974 *R*^2^: 0.897) represent an LD-block of 292.34 kb and reside in the introns of the LINC00351 gene. The result is mostly driven by the *p*-value attained in the TS meta-analysis and there is evidence of significant confounding caused by heterogeneity.

We summarize the results of the meta-analysis on Table 1. In Table 2, we provide the annotation we generated for each significant variant, and in Table 3 we provide the LD regions associated with the variants. The full results of the meta-analysis on the 489 tested SNPs are described in more detail in the Supplementary Material.

**Table 1:** **Significant results of the meta-analysis**.

**SNP**	**Chromosome**	**Position**	**Allele**	**TS**	**ADHD**	**Meta**	**Direction**	***I*^2^**	**Het *P*-value**
rs1866863	6	13336583	A	6.09E-04	1.51E-04	3.23E-07	–	0	0.9916
rs2705462	4	155648650	T	8.30E-04	5.09E-04	1.44E-06	–	0	0.875
rs2458304	6	13323663	A	3.74E-04	1.77E-03	2.54E-06	–	0	0.5818
rs17561798	4	98314790	A	3.80E-04	6.01E-03	9.89E-06	–	0	0.4226
rs1650137	12	104586532	A	4.01E-04	9.58E-03	1.76E-05	++	0	0.3702
rs2246417	21	45339588	T	2.54E-04	1.42E-02	1.95E-05	++	13.7	0.2816
rs9319159	13	85445305	T	1.15E-05	1.11E-01	3.77E-05	–	79.2	0.02837
rs13244651	7	10308596	T	5.03E-05	6.18E-02	4.11E-05	++	67.8	0.07797
rs1007021	9	2723657	A	5.36E-05	6.26E-02	4.38E-05	++	67.5	0.07919
rs7858600	9	102764080	A	2.26E-04	3.42E-02	5.30E-05	++	42.8	0.1859
rs7336083	13	85429252	A	9.49E-06	1.71E-01	6.88E-05	–	82.3	0.01756
rs17531553	7	10311703	A	9.40E-05	6.73E-02	7.08E-05	++	64.5	0.09348
rs1896373	10	52334599	T	3.46E-04	3.50E-02	7.46E-05	++	35.8	0.2121
rs1007022	9	2723761	A	8.85E-05	7.33E-02	7.68E-05	++	66	0.08647
rs11716445	3	49368662	A	7.95E-04	2.22E-02	8.01E-05	++	0	0.336
rs477897	4	168177763	A	3.10E-04	4.19E-02	8.65E-05	++	42.5	0.1874
rs4789936	17	78901892	T	7.12E-04	2.61E-02	8.92E-05	–	5.1	0.3047
rs2285084	4	2904558	A	8.07E-04	2.66E-02	1.00E-04	–	1	0.3149
rs7203818	16	49610387	A	4.03E-04	4.09E-02	1.01E-04	++	37.1	0.2075

*Here we report the p-values attained in each study, the combined p-value after the meta-analysis and the direction of the effect. Alongside these statistics, we also present Cochran's I^2^ value and the heterogeneity p-value for each SNP*.

**Table 2:** **Functional annotation of the significant SNPs of the meta-analysis**.

**SNP**	**Chromosome**	**Position**	***P*-value**	**Gene**	**Impact**	**Global Freq**	**EUR Freq**
rs1866863	6	13336583	3.23E-07	TBC1D7	Regulatory region	A:0.4583	G:0.3907
rs2705462	4	155648650	1.44E-06	GUCY1A3	Intergenic	T:0.2041	T:0.2893
rs2458304	6	13323663	2.54E-06	TBC1D7	Intron	A:0.4287	G:0.3598
rs17561798	4	98314790	9.89E-06	RAP1GDS1	Intron	G:0.0110	G:0.0398
rs1650137	12	104586532	1.76E-05	CHST11	Intron	A:0.2911	A:0.2038
rs2246417	21	45339588	1.95E-05	LINC00316	Intron	C:0.2428	C:0.1461
rs9319159	13	85445305	3.77E-05	LINC00351	Intron	T:0.3259	T:0.3797
rs13244651	7	10308596	4.11E-05	Intergenic	Intergenic	G:0.3724	G:0.4066
rs1007021	9	2723657	4.38E-05	KCNV2	Intron	A:0.1388	A:0.0696
rs7858600	9	102764080	5.30E-05	Intergenic	Intergenic	G:0.3694	A:0.4861
rs7336083	13	85429252	6.88E-05	LINC00351	Intron	A:0.3311	A:0.3668
rs17531553	7	10311703	7.08E-05	Intergenic	Intergenic	G:0.3746	G:0.4066
rs1896373	10	52334599	7.46E-05	Intergenic	Regulatory region	T:0.4994	T:0.4513
rs1007022	9	2723761	7.68E-05	KCNV2	Intron	A:0.0649	A:0.0567
rs11716445	3	49368662	8.01E-05	RHOA	Intron	A:0.03115	A:0.1024
rs477897	4	168177763	8.65E-05	ANXA10	Intron	G:0.1148	G:0.2256
rs4789936	17	78901892	8.92E-05	CEP295NL	Intron	T:0.4407	T:0.5219
rs2285084	4	2904558	1.00E-04	ADD1	Intron	G:0.2414	G:0.2087
rs7203818	16	49610387	1.01E-04	ZNF423	Intron	A:0.2169	A:0.1581

*Here we present the genes in which the SNPs are located, along with the frequency of the alleles in global and european populations, according to 1000 Genomes*.

**Table 3:** **LD-regions that are captured by the top SNPs in our study**.

**SNP**	**Chromosome**	**Region**	**Length (kb)**	**Gene(s)**	**eQTL**
rs11716445	3	48446237.50387873	1941.64	PLXNB1;…NPRL2	–
rs2705462	4	156557255.156603882	46.63	GUCY1A3	–
rs477897	4	168980017.169310018	330.00	ANXA10; DDX60; DDX60L	–
rs2285084	4	2836195.2961783	125.59	SH3BP2; ADD1; MFSD10; NOP14	–
rs1866863	6	13298395.13352581	54.19	TBC1D7	1.7e-10 (rs499818)
rs17531553	7	10255806.10359733	103.93	–	–
rs7858600	9	105517266.105544614	27.35	–	–
rs1007021	9	2699874.2758034	58.16	KCNV2	–
rs1896373	10	54068904.54116478	47.58	DKK1	2.18e-05 (rs1919459)
rs1650137	12	104977289.105016971	39.68	CHST11	–
rs7336083	13	85893863.86186202	292.34	–	–
rs7203818	16	49637407.49658524	21.12	ZNF423	–
rs4789936	17	76894415.76928600	34.19	TIMP2; LOC100653515	–
rs2246417	21	46744358.46760648	16.29	–	–

*Regions that are in linkage disequilibrium with the top SNPs, along with the range of genes residing in those regions and any linked known eQTLs. Genomic coordinates are in reference to the GRCh37 assembly*.

## 4. Discussion

This is the first study to identify shared genetic factors underlying TS and ADHD, two closely related and often co-occurring neuropsychiatric disorders (Karagiannidis et al., 2016). We meta-analyzed 489 of the top hit SNPs in the first TS GWAS, that had also been tested in ADHD published GWASs and meta-analysis. Our own meta-analysis highlights genes that may play a role in the shared etiology between TS and ADHD. 19 SNPs attained in the meta-analysis a *p*-value lower than the significance threshold, as denoted by the Bonferroni correction approach for multiple testing. All significant SNPs had the same direction of effect, which is indicative of a shared mechanism of disease development. A minority of those had not presented any association with ADHD in the original ADHD meta-analysis, with the resulting combined *p*-value being driven mostly by the *p*-value acquired from the TS study.

The five most significant SNPs had achieved moderate association *p*-values in the original ADHD study, and thus attained high *p*-values with no heterogeneity-based confounding in our meta-analysis, becoming strong candidates for the shared genomic background of the disorders.

TBC1D7 (TBC1 Domain Family, Member 7) is a prominent gene in our results, with two variants achieving the top and the third best *p*-values in our study. The top scoring SNP is located in a regulatory region downstream of the gene, while the third top is located within an intron of the gene. The associated variants have demonstrated linkage disequilibrium with a known eQTL for the expression of the gene, further substantiating their implication into the regulation of the expression profile of the gene. Expression profiling in Expression Atlas and GTEx show significant overexpression in the brain, the heart, the testis and in blood cells. The product of the gene is the third subunit of the TSC1-TSC2 complex with a Rheb-GAP activity, and is ubiquitously present in the complex (Dibble et al., 2012). An eQTL for TBC1D7 has been significantly associated with migraine and migraine without aura in a study of 23,285 individuals with migraine and 95,425 population-matched controls (Anttila et al., 2013). Interestingly, Anttila et al (Anttila et al., 2016) also picked up a genetic correlation between TS and migraine.

The presence of TBC1D7 in the TSC1/2 complex creates a suggestive functional link between the proteins. The role of the TSC1/2 complex is indicative of TBC1D7's role in the brain and neuropsychiatric disease, as an important component of the active complex. The TSC1 (Tuberous Sclerosis 1) and TSC2 (Tuberous Sclerosis 2) genes have an important role in the aetiology of Tuberous Sclerosis Complex (TSC). TSC is a neurodevelopmental disorder that typically presents with tumours of the brain, skin, heart, lungs, and kidneys, but also neurological disorders such as epilepsy, cognitive disability and autism. The TSC1/2 complex acts as an inhibitor of the mechanistic target of rapamycin (mTOR) signaling pathway which plays a central role in cell growth, proliferation, autophagy and thus also neurodevelopment (Henske et al., 2016). The TSC pathway regulates neuronal structure and function, and is sensitive to gene-dosage effects, showing degrees of haploinsufficiency (Tavazoie et al., 2005). TSC1 has also been implicated in bipolar disorder, without attaining genome-wide significance (Scott et al., 2009). Furthermore, TSC1 has been shown to have a neuroprotective role in hippocampal regions of the brain, protecting against ischemic events (Papadakis et al., 2013).

RAP1GDS1 (RAP1, GTP-GDP dissociation stimulator 1) is a GDP/GTP exchange protein with GTPase activity (Riess et al., 1993). It is located on chromosome 4 and is the third top locus to be implicated in the shared genetic background, with the associated variant residing in the intron of the gene. It is significantly overexpressed in brain and nervous tissues. RAP1GDS1 has been shown to interact with RHO (Ras homolog gene family, member A), that has also been implicated in this study, in a cascade involving interactions with multiple signaling proteins (Vikis et al., 2002; Berg et al., 2010; Hamel et al., 2011). CHST11 (carbohydrate chondroitin 4 sulfotransferase 11) is involved in the sulfation of chondroitin (Klppel, 2010), which is a key element of the brain matrix (Kwok et al., 2012). It is expressed in areas of the brain, including the hippocampus and the caudate nucleus. GUCY1A3 (Guanylate cyclase soluble subunit alpha-3) functions as the main receptor for nitric oxide, and has been implicated in Moyamoya disease, a disease causing constriction in arteries and brain ischemic events (Wallace et al., 2016).

## 5. Conclusion

We investigate, for the first time, the common genetic background between TS and ADHD on a genomewide scale and provide evidence that specific genes may underlie both disorders. The implicated variants lie on genes that appear to have a complex interplay between them. The main theme of the results is the Ras signaling cascade in the brain, with TBC1D7 and RAP1GDS1 being key elements of the brain signaling pathways. Interestingly, an additional theme emerging from the data, is related to brain ischemic response, with GUCY1A3 and the TSC1/2 complex (which includes TBC1D7) as implicated as factors. Intriguingly, one of our top hits, TBC1D7, implicates the mTOR signaling pathway and autophagy processes (Dibble et al., 2012). Furthermore, our analysis also points to CHST11, which has been shown to regulate the brain extracellular matrix, by affecting the chondroitin sulfation levels. Therefore, further investigation in the role of the respective genes in the shared genetic aetiology of TS and ADHD is warranted. Our results provide an intriguing insight into the shared mechanism of common neuropsychiatric disorders.

## Author contributions

FT, SP, JA, IK, MT, AT, DM, MG, PD performed data analysis and interpretation, and participated in manuscript writing. PP designed and supervised the study, performed data analysis and interpretation, and participated in manuscript writing. All authors read and approved the final version to be published and agreed to be accountable for all aspects of the work.

## Funding

This study was supported by the TS-EUROTRAIN and the EMTICS projects, funded by the European Union under the Seventh Framework People Programme (TS-EUROTRAIN, FP7-PEOPLE-2012-ITN, Grant Agr.No.316978 EMTICS, FP7-HEALTH, Grant Agr.No. 278367).

### Conflict of interest statement

The authors declare that the research was conducted in the absence of any commercial or financial relationships that could be construed as a potential conflict of interest. The handling Editor declared a current collaboration with one of the authors PP and states that the process nevertheless met the standards of a fair and objective review.
